# Texture features of periaqueductal gray in the patients with medication-overuse headache

**DOI:** 10.1186/s10194-017-0727-0

**Published:** 2017-02-02

**Authors:** Zhiye Chen, Xiaoyan Chen, Mengqi Liu, Shuangfeng Liu, Lin Ma, Shengyuan Yu

**Affiliations:** 1 Department of Radiology, Chinese PLA General Hospital, Beijing, 100853 China; 2 Department of Neurology, Chinese PLA General Hospital, Beijing, 100853 China; 3 Department of Radiology, Hainan Branch of Chinese PLA General Hospital, Beijing, 100853 China

**Keywords:** Brain, Magnetic resonance imaging, Medication-overuse headache, Migraine, Periaqueductal gray, Texture analysis

## Abstract

**Background:**

Periaqueductal gray (PAG) is the descending pain modulatory center, and PAG dysfunction had been recognized in migraine. Here we propose to investigate altered PAG texture features (quantitative approach for extracting texture descriptors for images) in the patients with medication-overuse headache (MOH) based on high resolution brain structural image to understand the MOH pathogenesis.

**Methods:**

The brain structural images were obtained from 32 normal controls (NC) and 44 MOH patients on 3.0 T MR system. PAG template was created based on the ICBM152 gray matter template, and the individual PAG segment was performed by applying the deformation field to the PAG template after structural image segment. Grey-level co-occurrence matrix (GLCM) was performed to measure the texture parameters including angular second moment (ASM), Contrast, Correlation, inverse difference moment (IDM) and Entropy.

**Results:**

Contrast was increased in MOH patients (9.28 ± 3.11) compared with that in NC (7.94 ± 0.65) (*P* < 0.05), and other texture features showed no significant difference between MOH and NC (*P* > 0.05). The area under the ROC curve was 0.697 for Contrast in the distinction of MOH from NC, and the cut-off value of Contrast was 8.11 with sensitivity 70.5% and specificity 62.5%. The contrast was negatively with the sleep scores (*r* = -0.434, *P* = 0.003).

**Conclusion:**

Texture Contrast could be used to identify the altered MR imaging characteristics in MOH in understanding the MOH pathogenesis, and it could also be considered as imaging biomarker in for MOH diagnosis.

**Electronic supplementary material:**

The online version of this article (doi:10.1186/s10194-017-0727-0) contains supplementary material, which is available to authorized users.

## Background

Periaqueductal gray (PAG) is a center with powerful descending pain modulatory center in the midbrain, which include various layered neurons around the aquaeductus mesencephali [1, 2], and whose dysfunction had been recognized in migraine [3]. PAG, as a substantial descending pain modulatory center, exerts inhibition and facilitation control on nociceptive transmission in the dorsal horn and trigeminal nucleus [4], and the modulatory mechanism was exerted by descending PAG-RVM (rostral ventromedial medulla) pathway contributing to central sensitization and development of secondary hyperalgesia [4, 5]. PAG included multiple types of neurons (eg. L-glutamate,γ-aminobutyric acid (GABA), opioids (particularly enkephalin), substance P), and had distinct connections with the forebrain, brainstem, and nociceptive neurons of lamina I of the spinal cord and trigeminal nucleus [6–9]. Therefore, PAG was confirmed as a critical component of a network responsing to pain and receiving functionally input from nociceptive pathways [10–12].

In the previous studies, the specific PAG lesions had been identified in multiple sclerosis [13–17] and infarction [18], and nonspecific PAG lesions was also revealed in episodic migraine (EM) patients in our previous study [19]. The specific lesions was the direct evidence for migraine, and the nonspecific lesions was indirectly used to explain the migraine pathogenesis, which may be associated with iron deposition and may be considered as a possible "generator" of migraine attacks [1, 20, 21]. However, the neuromechinism for nonspecific PAG lesions in migraine was still not elucidated up to now.

Medication-overuse headache (MOH) is a secondary form of chronic headache deriving from episodic migraine (EM) related to the overuse of triptans, analgesics and other acute headache medications [22–24]. Resting-state functional MRI (rs-fMRI) demonstrated altered functional connectivity was revealed in MOH, and suggested that MOH is associated with intrinsic brain network changes rather with macrostructural changes [23]. Voxel-based morphometry (VBM) recognized that increased gray matter in the midbrain presented in MOH [25]. Recently, some studies also confirmed an altered nucleus accumbens functional connectivity of motivational circuits [22] and abnormal connectivity between the PAG and other pain modulatory (frontal) regions in MOH, which were consistent with dysfunctional central pain control [26]. Although the functional and structural MRI recognized the PAG dysfunction in MOH patients, these methods did not presented the detailed changes of the intrinsic natures of PAG in MOH patients.

Texture features are the intrinsic properties of image and provide an efficient image classification to detect subtle alterations in the gray level distribution of an image [27]. Texture feature analysis had been widely applied in the brain tumor [28, 29], epilepsy [30, 31], muscular dystrophy [32], Attention-Deficit/Hyperactivity Disorder classification [33], and mild cognitive impairment [34]. However, MR imaging texture feature analysis was not applied in medication-overuse headache (MOH) so far.

In this study, we hypothesize MOH patients without T2-visible lesions may present altered texture features changes in MR structural images. To address this hypothesis, we prospectively obtained high resolution structural images from 44 MOH patients and 32 NCs without T2-visible lesions on the brain. Gray level co-occurrence matrix(GLCM) [35, 36] was used to calculate the texture parameters of PAG including angular second moment (ASM), Contrast, Correlation, inverse difference moment (IDM) and Entropy in the subjects, which would be used to detect the texture features change for PAG to elucidate the neuromechnism of PAG dysfunction in MOH pathogenesis.

## Methods

### Subjects

Written informed consent was obtained from all participants according to the approval of the ethics committee of the local institutional review board. Forty-four MOH patients and 32 normal controls were recruited from the International Headache Center, Department of Neurology, Chinese PLA General Hospital. All the following inclusion criteria should be fulfilled: 1) MOH refers to ICHD-III beta 8.2, and the definition of migraine refers to ICHD-III beta 1.1 and 1.2 [37]; 2) no migraine preventive medication used in the past 3 months; 3) age between 20 and 60 years; 4) right-handed; 5) absence of any chronic disorders, including hypertension, hypercholesterolemia, diabetes mellitus, cardiovascular diseases, cerebrovascular disorders, neoplastic diseases, infectious diseases, connective tissue diseases, other subtypes of headache, chronic pain other than headache, severe anxiety or depression preceding the onset of headache, psychiatric diseases, etc.; 6) absence of alcohol, nicotine, or other substance abuse; and 7) patient’s willingness to engage in the study. Thirty-two normal controls (NCs) were recruited from the hospital’s staff and their relatives. Inclusion criteria were similar to those of patients, except for the first two items, and NCs should never have had any primary headache disorders or other types of headache in the past year. The exclusion criteria for NC and MOH were the following: cranium trauma, illness interfering with central nervous system function, psychotic disorder, and regular use of a psychoactive or hormone medication. General demographic and headache information were registered and evaluated in our headache database. Additionally, we evaluated anxiety, depression, and cognitive function of all the participants by using the Hamilton Anxiety Scale (HAMA) [38], the Hamilton Depression Scale (HAMD) [39], and the Montreal Cognitive Assessment (MoCA) Beijing Version (www.mocatest.org). All the patients were given with the Visual Analogue Scale (VAS), migraine disability assessment (MIDSA), and a standard categorical four-grade sleep disturbance scale (SDS)(0, normal; 1, mild sleep disturbance; 2, moderate sleep disturbance; 3, serious sleep disturbance). MRI scans were taken in the interictal stage at least three days after a migraine attack for MOH patients. All the subjects were right-handed and underwent conventional MRI examination to exclude the subjects with cerebral infarction, malacia, or occupying lesions. Alcohol, nicotine, caffeine, and other substances were avoided for at least 12 h before MRI examination.

### MRI acquisition

Images were acquired on a GE 3.0 T MR system (DISCOVERY MR750, GE Healthcare, Milwaukee, WI, USA) and a conventional eight-channel quadrature head coil was used. All subjects were instructed to lie in a supine position, and formed padding was used to limit head movement. A three-dimensional T1-weighted fast spoiled gradient recalled echo (3D T1-FSPGR) sequence generating 180 contiguous axial slices [TR (repetition time) = 6.3 ms, TE (echo time) = 2.8 ms, flip angle = 15°, FOV (field of view) = 25.6 cm × 25.6 cm, Matrix = 256 × 256, NEX (number of acquisition) = 1] was used to perform the new segment and the individual PAG creation. Conventional T2-weighted imaging (T2WI), T1 fluid-attenuated inversion recovery (T1-FLAIR) and diffusion weighted imaging (DWI) were also acquired. All imaging protocols were identical for all subjects. No obvious structural damage and T2-visible lesion were observed on the conventional MR images.

### MR image processing

**Fig. 1:**
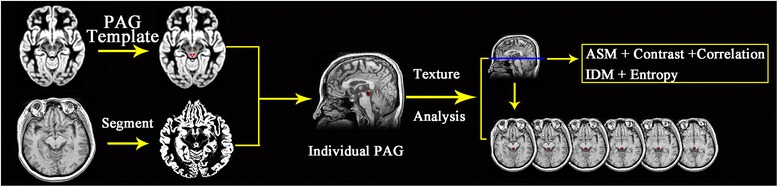
The flowchart of PAG texture calculation. GLCM, grey-level co-occurrence matrix; ASM, Angular Second Moment; IDM, Inverse Difference Moment

All MR structural image data were processed using Statistical Parametric Mapping 12 (SPM12) (http://www.fil.ion.ucl.ac.uk/spm/) running under MATLAB 7.6 (The Mathworks, Natick, MA, USA) to perform segment [40]. The image processing included following steps: (1) Create PAG template based on mni_icbm152_gm_tal_nlin_asym_09a template using MRIcron software (http://people.cas.sc.edu/rorden/mricron/index.html); (2) The structural images segment were performed with the new segment tool of SPM12 software, and the deformation field(iy_subjectid.nii) was generated. (3) Individual PAG mask (wPAG.nii) was generated by apply the deformation field (generated by new segment) to the PAG template using deformations tool of SPM12 software; (4) The individual PAG(wPAG_AddMask.nii) were segmented by an in-house script written on MATLAB (the Math Works, Inc., Natick, MA, USA) platform. The in-house script was provided in the in Additional file 1. (5) The PAG texture parameters were calculated over the whole PAG using gray-level co-occurrence matrix(GLCM) with the GLCM plugins on ImagJ(1.50i)(https://imagej.nih.gov/ij). The texture parameters included ASM, Contrast, Correlation, IDM and Entropy [35, 41] (Fig. 1). These texture parameters were measured on each slice, and the mean texture parameters values over all the slices were regarded as the final texture parameter value.

### Statistical analysis

The statistical analysis was performed by using PASW Statistics 18.0. Independent sample *T* test was applied to age, HAMA, HAMD, MoCA scores and all the texture parameters. Chi-Square test was applied to sex. Pearson correlation analysis was applied between Contrast and the clinical variables. Significant difference was set at a *P* value of < 0.05. Receiver operating characteristics (ROC) curve analysis was applied to evaluate the diagnostic efficacy of Contrast.

## Results

### Demography and neuropsychological test

There was no significant difference for age and sex between MOH and NC, a significant difference for HAMA between MOH(18.25 ± 8.74) and NC (10.19 ± 2.98), HAMD between MOH (19.80 ± 11.85) and NC (8.03 ± 4.34), MoCA among MOH (23.43 ± 3.72) and NC (27.16 ± 2.32) (Table 1).

**Table 1:** The clinical characteristics of normal controls and MOH patients

	NC	MOH	T value	*P* value
Sex(M/F)	32(12/20)	44(9/35)	2.692^a^	0.101
Age(year)	41.34 ± 10.89	42.30 ± 9.62	0.403	0.688
HAMA	10.19 ± 2.98	18.25 ± 8.74	5.002	0.000
HAMD	8.03 ± 4.34	19.80 ± 11.85	5.354	0.000
MoCA	27.16 ± 2.32	23.43 ± 3.72	4.999	0.000
DD(year)	11.25 ± 9.30			
VAS	7.88 ± 1.45			
MIDSA	101.81 ± 53.95			
Frequence(month)	24.81 ± 6.32			
SDS	2.23 ± 1.36			

^a^Chi-Square test; *NC* normal control, *MOH* medication-overuse headache, *DD* disease duration, *VAS* visual analogue scale; *HAMA* Hamilton Anxiety Scale, *HAMD* Hamilton Depression Scale, *MoCA* Montreal Cognitive Assessment, *NA* not available, *SDS* standard categorical four-grade sleep disturbance scale (0, normal; 1, mild sleep disturbance; 2, moderate sleep disturbance; 3, serious sleep disturbance)

### Comparison of PAG texture parameters between MOH and NC

**Fig. 2:**
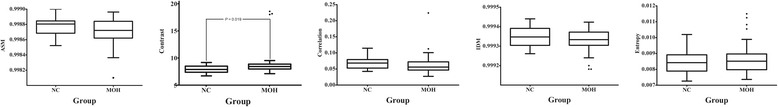
Comparison of PAG texture between MOH and NC, and the significant difference of Contrast presented in MOH compared with NC

Table 2 demonstrated that there was a significant increased Contrast in MOH (9.28 ± 3.11) compared with that in NC (7.94 ± 0.65)(*P* < 0.05). The ASM, Correlation, IDM and Entropy showed no significant difference between MOH and NC. Figure 2 presented the distribution of increased Contrast in MOH patients, and the other texture parameters showed no significant change in MOH patients compared with NC patients.

**Table 2:** Comparison of PAG texture parameters among NC and MOH

	NC	MOH	T value	*P* value
ASM(×10^-3^)	0.998768 ± 0.000124	0.998709 ± 0.000175	1.656	0.102
Contrast	7.943417 ± 0.645233	9.282469 ± 3.109250992	2.395	0.019
Correlation	0.06879277 ± 0.01808873	0.062252806 ± 0.030849871	1.072	0.287
IDM(×10^-3^)	0.999345 ± 0.000051	0.999331992 ± 0.0000595448	1.007	0.317
Entropy(×10^-5^)	0.008454 ± 0.000705	0.008718528 ± 0.00096365	1.317	0.192

*ASM* Angular Second Moment, *IDM* Inverse Difference Moment

### ROC curve analysis and correlation analysis for Contrast

**Fig. 3:**
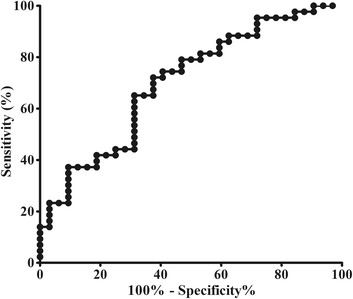
ROC curve for Contrast to diagnose MOH from NC. The area under the curve was 0.697, and the cut-off value of Contrast was 8.11 with sensitivity 70.5% and specificity 62.5%

ROC analysis demonstrated that area under curve (AUC) of Contrast was 0.697 in NC vs. MOH, and the cut-off value was 8.11 with sensitivity 70.5% and specificity 62.5% (Fig. 3). The contrast was negatively with the SDS scores (*r* = -0.434, *P* = 0.003), and presented no significant correlation with other clinical variables.

## Discussion

GLCM has proved to be a popular statistical method of extracting textural feature from MR images. In this study, five texture features were extracted, and previous study recognized that ASM represented the image energy, IDM represented the local homogeneity, Entropy represented the amount of information of the image that is needed for the image compression, and Correlation represented the linear dependency of grey levels of neighboring pixels [42]. In the current study, these four texture features did not showed significant difference between MOH patients and NCs, which suggested these four texture features could not elucidate the MOH pathogenesis, and they were not considered as diagnostic variables.

Contrast represents the amount of local gray level variation in an image, and a high value high value of this parameter may indicate the presence of noise or “wrinkled” textures in the image [30]. In this study, the increased Contrast texture parameter was identified in MOH patients, which suggested that PAG present increased local gray level variation in MR T1 images. The increased noise in PAG image may be associated with local heterogeneous intensity, which may be influenced by the iron deposition [20, 21] or other factors, and the neuromechanism should be further investigated.

Further ROC analysis revealed that AUC was 0.697 for Contrast, and the cut-off value (8.11) presented with sensitivity 70.5% and specificity 62.5%, which indicated that Contrast might be consider as an imaging biomarker for the diagnosis of MOH. Correlation analysis demonstrated that Contrast was negatively related to SDS scores, which indicated that decreased sleep quality was associated with the MOH pathogenesis. And other clinical variables showed no any correlation with Contrast, and these findings demonstrated that neuropsychological factors and pain intensity may be not associated with the PAG dysfunction in MOH pathogenesis.

In this study, GLCM method was used to calculate the MR image texture features on MOH patients, and texture Contrast was screened from the five texture parameters to explain the PAG dysfunction in MOH patients. However, there were several limitations in our study. Firstly, this study was based on GLCM method to calculate the texture features of PAG, and the other novel texture analysis methods such as histogram analysis and first order texture analysis should be considered in the future. Secondly, only five texture features were calculated in this study, and more texture features should be measured to screen the significant texture features for MOH patients. Lastly, 3D high resolution structural image was used to calculate the PAG texture features because of its high contrast for PAG, and the other MR images such as T2 weighted image and susceptibility weighted image should also be considered for the texture analysis in the future.

## Conclusion

In conclusion, this study revealed that altered PAG texture Contrast presented in MOH patients undetected by visual assessment, and it may be associated with PAG dysfunction and may be considered as a auxiliary diagnostic and evaluated imaging biomarker in MOH patients.

## Additional file

Additional file 1: The in-house script written on MATLAB (the Math Works, Inc., Natick, MA, USA) platform was used to segment individual PAG. (DOC 22 kb)

## Abbreviations

ASM: angular second moment
GLCM: grey-level co-occurrence matrix
IDM: inverse difference moment
MOH: medication-overuse headache
NC: normal controls
PAG: periaqueductal gray
