# Supplementary material for: Texture features of periaqueductal gray in the patients with medication-overuse headache
Source: J Headache Pain. 2017 Feb 2;18(1):14. doi: 10.1186/s10194-017-0727-0 (PMC5289934; doi:10.1186/s10194-017-0727-0)
Supplement: Additional file 1: — The in-house script written on MATLAB (the Math Works, Inc., Natick, MA, USA) platform was used to segment individual PAG. (DOC 22 kb) [file 10194_2017_727_MOESM1_ESM.doc]

cmDir='D:\PAG_MOH\MOH\MOH_CM_44_zcr\MOH_44.nii';

pagDir='D:\PAG_MOH\MOH\MOH_CM_44_zcr\wPAG.nii';

saveFile='D:\PAG_MOH\MOH\MOH_CM_44_zcr\wPAG_AddMask.nii';

cm=load_nii(cmDir);

pag=load_nii(pagDir);

cm.img(pag.img<1)=0;

save_nii(cm,saveFile);
